# Supplementary material for: Perception, regulation, and effects on longevity of pollen fatty acids in the honey bee, Apis mellifera
Source: PLoS One. 2024 Nov 21;19(11):e0309789. doi: 10.1371/journal.pone.0309789 (PMC11581215; doi:10.1371/journal.pone.0309789)
Supplement: S1 Text — Analysis of FA contents of honey bee collected pollen using gas chromatography- mass spectrometry. (DOCX) [file pone.0309789.s001.docx]

Fatty acid analysis:

To analyse the FA content of the honey bee collected pollen used in all experiments, the pollen was first ground into a fine powder using a coffee grinder (Bosch, Germany) and well mixed for homogenisation. In total two samples were analysed and the mean value for every FA was calculated.

For every sample, 0.53 – 0.54 mg of pollen was weighted into a 2 mL vial and 7 µL of FA standard (nonadecanoid acid in methanol, 0.2 mg/mL, Sigma-Aldrich, Saint Louis, MO, not found in pollen or other plant tissues) and 0.5 mL of a chloroform-methanol mix (2:1, Sigma-Aldrich, Saint Louis, MO) were added. To disrupt the pollen exines a glass rod was used until the pollen grains were nearly imperceptible in the solvent. The vial was then filled with the chloroform-methanol mixture to a final volume of 1.5 mL and transferred into a larger glass vial (with a minimum volume of 3 mL). The vial was then rinsed with an additional 1.5 mL of the chloroform-methanol mixture and the rinse was combined with the previously transferred solution in the larger glass vial.

Thereafter, the big glass vials were placed in a thermocycler and shaken for 24 hours at room temperature at 300 rpm. The next day, after the incubation period has completed and the pollen has settled down, excess solvent was evaporated under controlled nitrogen (N_2_) airflow until only a small amount of liquid was left. Subsequently, the supernatant was transferred into an inlet (300 µL) within the vial and the liquid was evaporated completely under the N_2_ air flow. Until further analysis, samples were stored at -20°C.

Prior to gas chromatography/mass spectrometry (GC-MS) analysis, 20 µL of Trimethylsulfonium hydroxide (TMSH, Macherey Nagel, Düren, Germany) was added as a fatty acid (FA) derivatization agent and FAs were derivatized into fatty acid methyl esters (FAMEs). This mixture was vortexed and then run in the GC-MS (GC: 6890N, MSD: 5975, Agilent Technologies, Santa Clara, United States).

To identify FAs, their mass spectra, retention times, and retention indices were analysed, and compared to the FA standard’s (nonadecanoic acid) area. The total FA content was obtained by summing up all FAs.

A control (blank sample run without pollen) was run to evaluate possible FA contamination accumulated during the process. If contamination was detected, the calculated concentration was subtracted from the sample profiles.
