# Supplementary material for: Perception, regulation, and effects on longevity of pollen fatty acids in the honey bee, Apis mellifera
Source: PLoS One. 2024 Nov 21;19(11):e0309789. doi: 10.1371/journal.pone.0309789 (PMC11581215; doi:10.1371/journal.pone.0309789)
Supplement: S1 Table — Results of TukeyHSD post-hoc test analyzing differences in pollen (white background) and FA consumption (grey background) between diets enriched with low, medium and high FA concentrations for the first seven days. (DOCX) [file pone.0309789.s002.docx]

**S1 Table: Differences in pollen consumption and FA consumption.**

|  |  | **Control diet** | **Low FA diet** | **Medium FA diet** | **High FA diet** |
| --- | --- | --- | --- | --- | --- |
| **1^st^ week** | **Control diet** |  | *P*=0.221 | ***P*<0.001** | ***P*<0.001** |
|  | **Low FA diet** | *P*=0.179 |  | ***P*<0.001** | ***P*<0.001** |
|  | **Medium FA diet** | ***P*<0.001** | ***P*<0.001** |  | ***P*<0.001** |
|  | **High FA diet** | ***P*<0.001** | ***P*<0.001** | *P*=0.999 |  |
|  | | | | | |
| **2^nd^ week** | **Control diet** |  | *P*=0.987 | *P*=0.62 | *P*=0.115 |
|  | **Low FA diet** | ***P*<0.001** |  | *P*=0.138 | *P*=0.231 |
|  | **Medium FA diet** | ***P*<0.001** | *P*=0.307 |  | *P*=0.994 |
|  | **High FA diet** | ***P*<0.001** | *P*=0.995 | *P*=0.444 |  |
|  |  | | | | |
| **3^rd^ week** | **Control diet** |  | *P*=0.986 | *P*=0.814 | *P*=0.108 |
|  | **Low FA diet** | *P*=1 |  | *P*=0.608 | ***P*=0.047** |
|  | **Medium FA diet** | ***P*<0.001** | ***P*<0.001** |  | *P*=0.516 |
|  | **High FA diet** | *P*=1 | *P*=1 | ***P*<0.001** |  |

Results of TukeyHSD post-hoc test analyzing differences in pollen (white background) and FA consumption (grey background) of *A. mellifera* workers between diets enriched with low, medium and high FA concentrations for every week separated over the duration of the experiment. Significant values are depicted in bold.
