# Supplementary material for: Perception, regulation, and effects on longevity of pollen fatty acids in the honey bee, Apis mellifera
Source: PLoS One. 2024 Nov 21;19(11):e0309789. doi: 10.1371/journal.pone.0309789 (PMC11581215; doi:10.1371/journal.pone.0309789)
Supplement: S2 Table — All FAs were solved 1:1000 in chloroform. Each stimulus was tested as CS+ as well as CS- to test whether the rewarded stimulus influenced learning performance. If this was the case, data was not pooled but analyzed separately, otherwise it was pooled. To test if the bees can differentiate between two stimuli a paired Wilcoxon signed-rank test with continuity correction was used. N represents the number of bees tested per stimulus pair. (DOCX) [file pone.0309789.s003.docx]

**Table S2: Results of the chemotactile PER conditioning experiments.**

| **Stimulus pair** | **U** | **N** | ***P*** |
| --- | --- | --- | --- |
| Omega-3 vs CHCl_3_ | U_31_=2 | 32 | ***P*<0.0001** |
| Omega-6 vs CHCl_3_ | U_32_=15 | 32 | ***P*<0.0001** |
| Omega-9 vs CHCl_3_ | U_30_=2.5 | 31 | ***P*<0.0001** |
| Capric acid (CS+) vs CHCl_3_ | U_15_=0 | 16 | ***P*<0.001** |
| Capric acid vs CHCl_3_ (CS+) | U_15_=0 | 16 | ***P*<0.001** |
| Stearic acid vs CHCl_3_ | U_31_=97 | 32 | *P*=0.127 |
| Omega-3(CS+) vs omega-6 | U_15_=0 | 16 | ***P*<0.001** |
| Omega-3 vs omega-6 (CS+) | U_15_=2 | 16 | ***P*<0.001** |
| Omega-3 vs omega-9 | U_30_=8 | 31 | ***P*<0.0001** |
| Omega-3 (CS+) vs capric acid | U_14_=0 | 15 | ***P*<0.001** |
| Omega-3 vs capric acid (CS+) | U_14_=2 | 15 | ***P*<0.001** |
| Omega-3 vs stearic acid | U_31_=110 | 32 | *P*=0.0575 |
| Omega-6 (CS+) vs omega-9 | U_15_=0 | 16 | ***P*<0.001** |
| Omega-6 vs omega-9 (CS+) | U_15_=2.5 | 16 | ***P*<0.005** |
| Omega-6 (CS+) vs capric acid | U_15_=1 | 16 | ***P*<0.005** |
| Omega-6 vs capric acid (CS+) | U_12_=1 | 13 | ***P*<0.005** |
| Omega-6 vs stearic acid | U_15_=16 | 30 | ***P*<0.001** |
| Omega-9 (CS+) vs capric acid | U_15_=0 | 16 | ***P*<0.001** |
| Omega-9 vs capric acid (CS+) | U_15_=0 | 16 | ***P*<0.005** |
| Omega-9 vs stearic acid | U_30_=4 | 31 | ***P*<0.0001** |
| Capric acid vs stearic acid | U_31_=2.5 | 32 | ***P*<0.0001** |
| Mix 1:100 (CS+) vs CHCl_3_ | U_15_=0 | 16 | ***P*<0.001** |
| Mix 1:100 vs CHCl_3_ (CS+) | U_15_=0 | 16 | ***P*<0.001** |
| Mix 1:1 000 (CS+) vs CHCl_3_ | U_14_=1.5 | 15 | ***P*<0.005** |
| Mix 1:1 000 vs CHCl_3_ (CS+) | U_15_=10.5 | 16 | ***P*<0.01** |
| Mix 1:10 000 (CS+) vs CHCl_3_ | U_15_=22 | 16 | ***P*<0.02** |
| Mix 1:10 000 vs CHCl_3_ (CS+) | U_15_=13.5 | 16 | *P*=0.159 |
| Mix 1:100 (CS+) vs Mix 1:1 000 | U_14_=2 | 15 | ***P*<0.01** |
| Mix 1:100 vs Mix 1:1 000 (CS+) | U_15_=9 | 16 | ***P*<0.02** |

All FAs were solved 1:1000 in chloroform. Each stimulus was tested as CS+ as well as CS- to test whether the rewarded stimulus influenced learning performance. If this was the case, data was not pooled but analyzed separately, otherwise it was pooled. To test if the bees can differentiate between two stimuli a paired Wilcoxon signed-rank test with continuity correction was used. N represents the number of bees tested per stimulus pair.
