# Supplementary figures and images for: Perception, regulation, and effects on longevity of pollen fatty acids in the honey bee, Apis mellifera
Source: PLoS One. 2024 Nov 21;19(11):e0309789. doi: 10.1371/journal.pone.0309789 (PMC11581215; doi:10.1371/journal.pone.0309789)

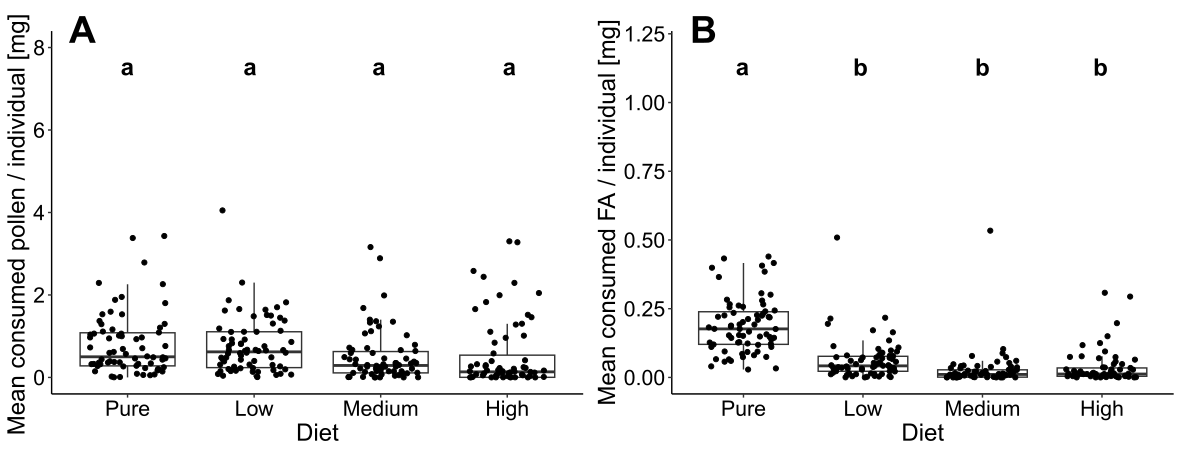

Supplement: S1 Fig — Mean consumption of (A) pollen and (B) FAs per individual and day during day seven to 14. Bees were fed with four different pollen diets differing in FA content (i.e. pure honey bee collected pollen as a control and the same pollen enriched with a FA-mix to achieve low FA pollen (1.5 times higher FA concentration), medium FA pollen (5 times higher FA concentration) and high FA pollen (10 times higher FA concentration)). Different letters above boxplots indicate significant differences in the mean consumption of pollen. (TIF) [file pone.0309789.s004.tif]

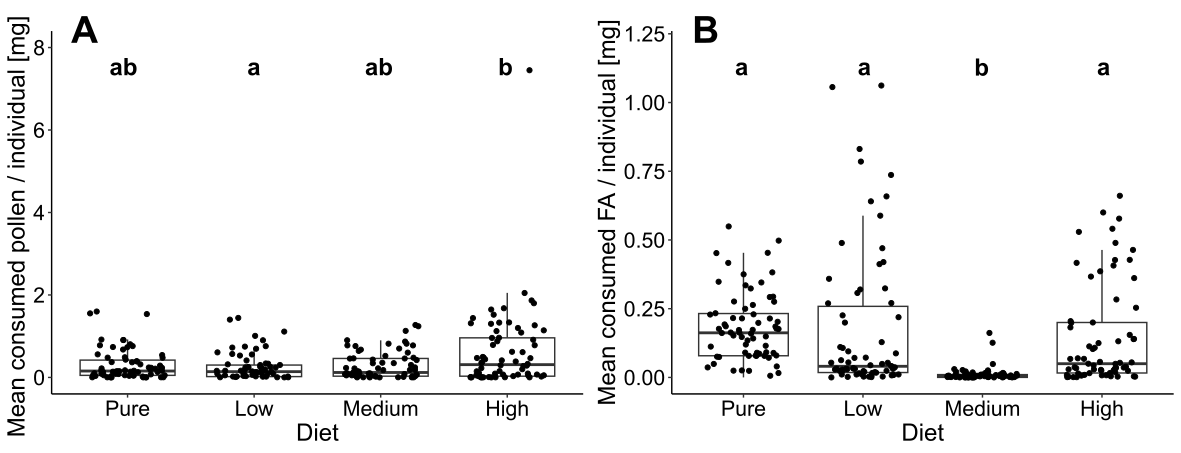

Supplement: S2 Fig — Mean consumption of (A) pollen and (B) FAs per individual and day during day 15 to 21. Bees were fed with four different pollen diets differing in FA content (i.e. pure honey bee collected pollen as a control and the same pollen enriched with a FA-mix to achieve low FA pollen (1.5 times higher FA concentration), medium FA pollen (5 times higher FA concentration) and high FA pollen (10 times higher FA concentration)). Different letters above boxplots indicate significant differences in the mean consumption of pollen. (TIF) [file pone.0309789.s005.tif]

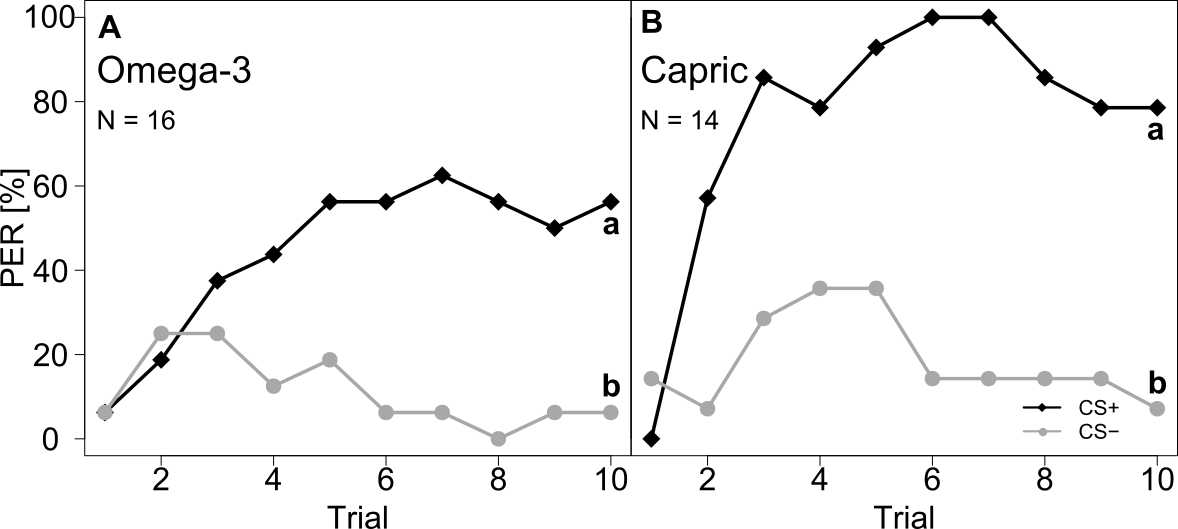

Supplement: S3 Fig — (A) Omega-3 was used as the rewarded stimulus and capric acid as the unrewarded stimulus. (B) Capric acid was used as rewarded stimulus and omega-3 as unrewarded stimulus. CS+ (black, squares) represents the rewarded stimulus and CS- (grey, circles) the unrewarded stimulus. N represents the number of individuals tested. Statistical differences are marked with different letters at the right side of the curves (P < 0.05). (TIF) [file pone.0309789.s006.tif]
