# Supplementary material for: Perception, regulation, and effects on longevity of pollen fatty acids in the honey bee, Apis mellifera
Source: PLoS One. 2024 Nov 21;19(11):e0309789. doi: 10.1371/journal.pone.0309789 (PMC11581215; doi:10.1371/journal.pone.0309789)
Supplement: Attachment — Submitted filename: Response to Reviewers_final.docx [file pone.0309789.s007.docx]

**From the Academical Editor:**

Dear Dr. Leonhardt,

Thank you for submitting your manuscript to PLOS ONE. After careful consideration, we feel that it has merit but does not fully meet PLOS ONE’s publication criteria as it currently stands. Therefore, we invite you to submit a revised version of the manuscript that addresses the points raised during the review process.

Please submit your revised manuscript by Jul 25 2024 11:59PM. If you will need more time than this to complete your revisions, please reply to this message or contact the journal office at [plosone@plos.org](mailto:plosone@plos.org). When you're ready to submit your revision, log on to <https://www.editorialmanager.com/pone/> and select the 'Submissions Needing Revision' folder to locate your manuscript file.

Please include the following items when submitting your revised manuscript:

- A rebuttal letter that responds to each point raised by the academic editor and reviewer(s). You should upload this letter as a separate file labeled 'Response to Reviewers'.
- A marked-up copy of your manuscript that highlights changes made to the original version. You should upload this as a separate file labeled 'Revised Manuscript with Track Changes'.
- An unmarked version of your revised paper without tracked changes. You should upload this as a separate file labeled 'Manuscript'.

If you would like to make changes to your financial disclosure, please include your updated statement in your cover letter. Guidelines for resubmitting your figure files are available below the reviewer comments at the end of this letter.

If applicable, we recommend that you deposit your laboratory protocols in protocols.io to enhance the reproducibility of your results. Protocols.io assigns your protocol its own identifier (DOI) so that it can be cited independently in the future. For instructions see: https://journals.plos.org/plosone/s/submission-guidelines#loc-laboratory-protocols. Additionally, PLOS ONE offers an option for publishing peer-reviewed Lab Protocol articles, which describe protocols hosted on protocols.io. Read more information on sharing protocols at https://plos.org/protocols?utm_medium=editorial-email&utm_source=authorletters&utm_campaign=protocols.

We look forward to receiving your revised manuscript.

Kind regards,

Olav Rueppell
Academic Editor
PLOS ONE

*Dear Editor,*

*Thank you very much for the possibility to submit a revised version of our manuscript to PLOS ONE. We also thank the two reviewers for their valuable and constructive comments on the manuscript. We have addressed all points raised by the reviewers and provide a detailed response to each comment individually below (starting with --).*

**Reviewer #1:** The manuscript by Schleifer et al. examines the ability of honey bees to detect the various fatty acids that are present in pollen, their ability to regulate this and the potential fitness effects. The manuscript is very interesting and adds to the existing literature of how honey bees are able to regulate nutrient intake, including fatty acids.

*-- Thank you very much for this remark, and also for taking the time to review our manuscript and for providing constructive feedback.*

My general comments are:
1. Please mention the full form before the use of abbreviations throughout the manuscript.

*-- Done.*

2. Replace animals with colonies in L114.

*-- Done.*

3. L120 why not use multiple colonies and multiple frames to avoid colony specific behavior and physiological results?

*-- Thank you for pointing that out, and we agree that this would be the optimal procedure. Unfortunately, at the time period of the experiment, we had only one colony with one mature brood frame available. Since this single brood frame had a sufficient number of bees emerged on the same day, we decided to stick with only one brood frame from a single colony. We now clarified this aspect in the manuscript and discuss the limitations of using only one colony.*

4. The total experimental duration for the feeding experiment should be included in the results. I found this eventually when reading the figure legends.

*-- The experimental duration is mentioned in the first sentence of the ‘Feeding experiment’ section (L127), but for clarification, we now also added the duration in the ‘Results’ section.*

5. How was FA concentration measured in pollen and throughout the experiment? A whole section is missing on how the authors evaluated the FA concentrations. Was mass spec used and where is the raw data?
*-- Thank you for pointing this out! We added the information of the FA analysis to the ‘Material & Method’ section and included a detailed protocol in the Supplementary Material section!*

6. L146 is there a physiological difference between honey bee foragers and their PER due to the long experimental duration and changing months?

*-- We could see differences in the response of foragers during the season. For example, early in the season, when many flowering plants were available, less bees were willing to show a PER when touching the antenna during the pre-test, compared to later in the season. However, only bees that showed a proper PER and thus a high motivational state, were used in the experiments. We additionally tested all stimuli randomly throughout the season to avoid any bias.*

7. What about the ages of the foragers? How was forager age controlled for?

*-- We randomly caught the bees while leaving the hive, which is why we could not control for age. However, studies showed, that honey bees’ cognitive abilities are influenced by their social role rather than the chronological age. We have added this information in the manuscript.*

8. More clarity in the method section is needed for better understanding the methods and for the benefit of the readers. L 148 were sister queens used? What was the total number of hives eventually used? Were the same newly emerged honey bees tested for all the experimental replicates? How many were tested for each group?

*-- In total, we used bees from ten hives (L158), but no sister queens were used.*

*Newly emerged bees were only used for the feeding experiment since the consumption of pollen by adult bees declines rapidly after the first week. Each bee was assigned to only one dietary treatment. We had 4 treatments with 10 replicates each. Each replicate comprised 30 newly emerged bees – so 300 bees for each dietary treatment (L131-133). Bees used in the feeding experiments were not used for the learning / PER experiments; only foragers that were about to leave the hive, were used for PER experiments. All this information has now been added/clarified in the methods.*

9. Similar to the experimental duration, methods do not indicate that chloroform was tested as a solvent control for PER. I found this later on in the manuscript. Please include this to help improve the method section for further clarity.
*-- Thank you for the hint! We now added this information to the ’Stimuli’ paragraph of the ‘Material and Method’ section.*

10. L230 for the benefit of the readers the authors should indicate how they calculated FA consumption data.

*-- Good point. This is now included in the beginning of the ‘Statistical analysis’ section.*

11. Please add the weekly data for the entire experimental duration even though the first week showed most consumptions. It will be interesting to see what happens every week. This will also add more information on how much pollen was consumed for each experimental group. And how much FA was also consumed.

*-- We agree and have now added the respective figures in the Supplementary Material*

12. L252 please italicize scientific names.

*-- Done.*

13. L254 the graph shown does not seem to match 21 days for experimental duration based on the axis.

*-- Thank you for the hint. We have revised the figure.*

14. For the PER experiments in general, are honey bees not trained with a sugar reward as a positive stimulus response and no sugar reward as a negative response? So it is expected that honey bees will learn and not respond when sugar reward is not presented? Even if FA were presented? Also was the same honey bee tested for all experimental groups in all combinations (solvent control and each FA as CS+ and CS-)? If not how were honey bees age controlled/colony controlled to negate any other bias? How many training attempts and how many test attempts for each honey bee?
*-- You are correct, the rewarded stimulus (CS+) is combined with sugar water (e.g. fatty acid A + sugar water), and the unrewarded stimulus (CS-) is not paired with anything but the stimulus alone (e.g. fatty acid B).
Naïve, untrained bees will only elicit a PER towards sugar water and not towards a FA alone. In a classical absolute conditioning experiment, when the FA is paired with the sugar water, the bee associates the sugar reward with this FA after repeated presentations and will elicit a PER to the FA alone, but only if the bee can perceive the FA. In a classical differential conditioning experiment, one FA will be rewarded (CS+), a second FA will be unrewarded (CS-). After repeated random presentations of the CS+ and CS-, the bee will elicit a PER only to the rewarded one, but no PER to the unrewarded one.
Regarding testing: an individual bee was tested only once (described in L184), otherwise the bee would already have learned one stimulus and cannot be considered a naïve, untrained bee.*

*We randomly took bees from different colonies to exclude any colony bias, but we did not check for age (although leaving forager bees were considered to be between 16 and 35 day old). Each bee was tested ten times for the rewarding and ten times for the unrewarded stimulus, in total 20 trials (L 196-197).*

*We have tried to clarify these details throughout the manuscript.*

15. All raw data have not been presented and please include that.

*-- The raw data for the FA analysis of the pollen has now been added and is available on https://doi.org/10.17605/osf.io/geuzv.*

16. While survival is a measure of fitness, not sure if the authors can truly use “fitness” in their manuscript title without actually measuring the various indices of fitness for honey bees (physiology and colony health).

*-- We agree with the reviewer and have changed the title to: Perception, regulation, and fitness effects on longevity of pollen fatty acids in the honey bee, Apis mellifera*

**Reviewer #2:** The authors should make a few changes before the document is forwarded to reviewers.

1. please check the reference format in the overall manuscript.
*-- Thank you for this hint; we checked for all mistakes and have now a consistent reference format.*

2. I think chloroform is toxic to honey bees. Did you check the safety of this chemical for honey bees? We attached the PDF which is chloroform MSDS

*-- This is correct; however, we have made sure that the chloroform evaporates completely before the filter paper was presented to the bees. We now added this important information to the Method section.*

3. According to the PER experiment, except for the combination of omega-3 and stearic acid, honeybees were able to distinguish each fatty acid. However, concluding that honeybees distinguish between saturated and unsaturated fatty acids seems unreasonable.

*-- We agree that our conclusion may have been too far-fetched and have adjusted our wording in the revised version (L358-359).*

4. Line 105: Please change ‘overconsumption’ to ‘High amount of FA content’.

*-- Done.*

5. Line 128: "0.5 times" means a decrease, not an increase. Therefore, please change all instances of 0.5 to 1.5.

*-- Done.*

6. Line 132: Please explain more clearly about the FA mixture, for example, where is the source (which company?) or Did you mix yourself using each FAs then, please list the company of each FA, for example: palmitic (Sigma-Aldrich, St. Louis, MO).

*-- Thank you for the hint. We added the source of the single FAs.*

7. Line 142: In Table 1, How did you measure the fatty acids content in pollen? And what is FS? Maybe FS should be changed to FA.

*-- Thank you for pointing this out. Of course, FS should be stated as FA, which we have changed now. We now also mention the FA analysis in the Material and Methods section and added the full protocol in the Supplementary Material.*

8. Line 237: Please add statistical results in Figure 2 (A and B). Please add the statistical result in the manuscript even present in the S1 Table.

*-- Done.*

9. Line 252: Please check that 'Apis mellifera' is in italics in all manuscripts.

*-- Done.*

10. Line 264: In Figure 3, please explain why CS- in stearic acid showed higher than other FAs.

*-- We do not know why the bees did not perceive stearic acid, which we mention and discuss in the text.*

11. Line 268: Why did you choose to PER test linoleic, linolenic, oleic, capric, and stearic acids among the fatty acids?
*-- We added our rationale to the Material and Method section, which now reads: “We selected these five FA to test if the bees can differentiate between FAs based on the chain length, saturation state or the number of double bounds”.*

12. Line 271: Please check that 'P' is in italics in all manuscripts.

*-- Done.*

13. Line 278: In Figure 4, Please list the full name of FAs, for example, ‘Omega-6 Capric’ change to ‘Omege-6 vs. Capric acid’. Please upgrade Figure 4.

*-- Done.*

14. Line 291: Please change ‘1:1000 vs chloroform’ to ‘1:1000 FA mix vs chloroform’.

*-- Done.*

15. In the discussion, please add more explanation of the percentage of each fatty acid (except Omega-3, Omega-6, and Omega-9) included in general pollen and explain the effect on honeybees.

*-- Thank you for mentioning it. We have added this information in the discussion*

16. Line 300: Please delete the second author name.

*-- Done.*

17. Line 305, 323, 332: Please delete the author's name. *-- Done.*
