# Supplementary material for: Perception, regulation, and effects on longevity of pollen fatty acids in the honey bee, Apis mellifera
Source: PLoS One. 2024 Nov 21;19(11):e0309789. doi: 10.1371/journal.pone.0309789 (PMC11581215; doi:10.1371/journal.pone.0309789)
Supplement: Attachment — Submitted filename: Response to Editor_2nd revision.docx [file pone.0309789.s008.docx]

Dear Dr. Leonhardt,

Thank you for re-submitting your manuscript to PLOS ONE. As you can see, the reviewer (and I) are satisfied with your revisions. However, before I can fully accept it, please fix the references at this stage. You might also want to give the manuscript an overall read through for catching minor errors. Finally, if you could please explain the authorship change?

Please submit your revised manuscript by Sep 26 2024 11:59PM. If you will need more time than this to complete your revisions, please reply to this message or contact the journal office at [plosone@plos.org](mailto:plosone@plos.org). When you're ready to submit your revision, log on to <https://www.editorialmanager.com/pone/> and select the 'Submissions Needing Revision' folder to locate your manuscript file.

Please include the following items when submitting your revised manuscript:

- A rebuttal letter that responds to each point raised by the academic editor and reviewer(s). You should upload this letter as a separate file labeled 'Response to Reviewers'.
- A marked-up copy of your manuscript that highlights changes made to the original version. You should upload this as a separate file labeled 'Revised Manuscript with Track Changes'.
- An unmarked version of your revised paper without tracked changes. You should upload this as a separate file labeled 'Manuscript'.

If you would like to make changes to your financial disclosure, please include your updated statement in your cover letter. Guidelines for resubmitting your figure files are available below the reviewer comments at the end of this letter.

If applicable, we recommend that you deposit your laboratory protocols in protocols.io to enhance the reproducibility of your results. Protocols.io assigns your protocol its own identifier (DOI) so that it can be cited independently in the future. For instructions see: [https://journals.plos.org/plosone/s/submission-guidelines#loc-laboratory-protocols](https://c05y1x9s.r.us-east-2.awstrack.me/L0/https:%2F%2Fjournals.plos.org%2Fplosone%2Fs%2Fsubmission-guidelines%23loc-laboratory-protocols/1/010f0191461c9ad8-3596a157-83f0-4336-a097-a31b2880882d-000000/G4uYCfVxh7G7BFNeY-YRy8lsx5g=171). Additionally, PLOS ONE offers an option for publishing peer-reviewed Lab Protocol articles, which describe protocols hosted on protocols.io. Read more information on sharing protocols at [https://plos.org/protocols?utm_medium=editorial-email&utm_source=authorletters&utm_campaign=protocols](https://c05y1x9s.r.us-east-2.awstrack.me/L0/https:%2F%2Fplos.org%2Fprotocols%3Futm_medium=editorial-email%26utm_source=authorletters%26utm_campaign=protocols/1/010f0191461c9ad8-3596a157-83f0-4336-a097-a31b2880882d-000000/11-raxe9CT5qhBKmg4D2wKyMfcU=171).

We look forward to receiving your revised manuscript.

Kind regards,

Olav Rueppell
Academic Editor
PLOS ONE

Journal Requirements:

Please review your reference list to ensure that it is complete and correct. If you have cited papers that have been retracted, please include the rationale for doing so in the manuscript text, or remove these references and replace them with relevant current references. Any changes to the reference list should be mentioned in the rebuttal letter that accompanies your revised manuscript. If you need to cite a retracted article, indicate the article’s retracted status in the References list and also include a citation and full reference for the retraction notice.

**Response:**

*Dear Dr. Rueppell,*

*Thank you very much for accepting our revised version of our manuscript to PLOS ONE. We also thank Hyung Wook Kwon for his valuable feedback on the manuscript. We have changed the reference list and added the missing information. The change in authorship is due to a mistake during the first submission of the manuscript, which we have now corrected. We apologize for the inconveniences. We also corrected some minor errors.*

*Kind regards,*

*Sara Leonhardt (on behalf of all co-authors)*
